# Envelope protein gene based molecular characterization of Japanese encephalitis virus clinical isolates from West Bengal, India: a comparative approach with respect to SA14-14-2 live attenuated vaccine strain

**DOI:** 10.1186/1471-2334-13-368

**Published:** 2013-08-08

**Authors:** Arindam Sarkar, Avishek Banik, Bani K Pathak, Subhra K Mukhopadhyay, Shyamalendu Chatterjee

**Affiliations:** 1 ICMR virus unit, GB- 4, 1st Floor, ID & BG Hospital, 57, Dr. S. C. Banerjee Road, Beliaghata, Kolkata 700010, West Bengal, India; 2 Department of Microbiology, The University of Burdwan, Golapbag, Burdwan, West Bengal, India; 3 Department of Biotechnology, St. Xavier’s College, Kolkata, West Bengal, India

**Keywords:** Japanese encephalitis virus, Envelope protein gene, Molecular phylogeny, Genotype III, Genotype I, Homology modeling, Hydrophilicity, T-cell epitope, West Bengal

## Abstract

**Background:**

Increasing virulence of Japanese encephalitis virus (JEV), a mosquito-borne zoonotic pathogen is of grave concern because it causes a neurotrophic killer disease Japanese Encephalitis (JE) which, in turn, is responsible globally for viral acute encephalitis syndrome (AES). Despite the availability of vaccine, JE/AES cases and deaths have become regular features in the different rural districts of West Bengal (WB) state, India, indicating either the partial coverage of vaccine or the emergence of new strain of JEV. Therefore, a study was undertaken to characterize and compare the complete envelope (E) protein gene based molecular changes/patterns of JEVs circulating in WB.

**Methods:**

Total of 98 AES case-patients’ samples were tested to detect the presence of JEV specific immunoglobulin M (IgM) antibody by Mac-ELISA method. Only JEV IgM negative samples with a history of ≤3 days’ illness were screened for virus isolation and RT-PCR. E gene sequences of JEV isolates were subjected to molecular phylogeny and immunoinformatics analysis.

**Results:**

Present study confirmed JEV etiology in 39.7% and 29.1% of patients presenting ≤15 days’ febrile illness, as determined by Mac-ELISA and RT-PCR respectively. Phylogenetic analysis based on complete E gene sequences of JEV isolates showed the co-circulation of JEV genotype I (GI) with genotype III (GIII). This study also demonstrated that isolate-specific crucial amino acid substitutions were closely related to neurovirulence/neuroinvasiveness of JE. On the basis of immunoinformatics analysis, some substitutions were predicted to disrupt T-cell epitope immunogenicity/antigenicity that might largely influence the outcome of vaccine derived from JEV GIII SA14-14-2 strain and this has been observed in a previously vaccinated boy with mild JE/AES due to JEV GI infection.

**Conclusions:**

Based on molecular evolutionary and bioinformatic approaches, we report evolution of JEV at a local level. Such naturally occurring evolution is likely to affect the disease profile and the vaccine efficacy to protect against JEV GI may demand careful evaluation.

## Background

The flaviviruses persist as causative agents for a wide range of human related infectious diseases worldwide. However, the most common causes of Acute Encephalitis Syndrome (AES) are associated with the Japanese encephalitis virus (JEV) serocomplex that includes Murray Valley encephalitis virus (MVEV), West Nile virus (WNV), St. Louis encephalitis virus (SLEV), and prototypical member JEV within/belonging to the *Flavivirus* genus under the family *Flaviviridae*[1,2]. The mosquito-borne JEV is the sole etiologic agent of Japanese encephalitis (JE); a neurotropic killer disease being one of the major causes of viral acute encephalitis in human. Since the isolation of this virus in Japan in 1935 [3], it has been detected worldwide becoming a major public health problem. Worldwide case-fatality rate of JE was recorded to be 30% approximately with 30-50% of survivors having irreversible neuropsychiatric sequelae [4].

The natural transmission cycle of JEV involves an enzoonotic (sylvatic) mosquito-bird-mosquito and/or mosquito-pig-mosquito cycle; primarily involving *Culex* spp. mosquitoes as primary vectors [5], wading birds as reservoir host [6], pigs as amplifying host [7] and Humans are the accidental “dead end” hosts [8].

Like other flaviviruses, JEV, an enveloped positive-sense single stranded RNA (~ 11 kb in length) virus contains single open reading frame (ORF) encoding a polyprotein that is processed into three structural (C, M, and E) and seven nonstructural (NS1, NS2A, NS2B, NS3, NS4A, NS4B, and NS5) proteins, flanked by 5′- and 3′-non-translated regions (NTRs) [9-11]. Among the three structural proteins, the envelope (E) protein is considered as the most antigenic part of the viral genome and is found to be involved in the majority of the biological properties of the virus, such as binding to the cell receptor, inducing immunological responses (neutralization, passive protection and antibody dependent enhancement), virion assembly and fusion activity at low pH [12,13]. In addition, the amino acid substitutions in E protein have a major role in determining the neuorovirulence or neuroinvasiveness [14]. The nucleotide sequence of full-length E gene of JEV is an established/reliable phylogenetic marker because this region has got no selective pressure supporting obscure long-term evolutionary relationship. Based on the nucleotide sequence of E gene, JEV can be divided into five distinct genotypes [15]. Mostly genotype III (GIII) is circulated in the Southeast Asian countries, including Japan, South Korea, China, Taiwan, Vietnam, Philippines, and India [2]. However, it has been recently documented that GIII is replaced by genotype I (GI) in South Korea, Thailand and China [16].

In India, the existence of JEV was first reported serologically in 1954 [17]. However, the disease was first recognized in India at Vellore (in the state of Tamil Nadu) in 1955 [18]. Since then, epidemics of JE in different states have been recorded [19,20]. It was mentioned that genotype III is predominant in India, but recently genotype I has been introduced in this country [16,21].

The state of West Bengal (WB) is situated at the eastern part (23°00′N, 87°00′E) of India with an area and population of 88,752 km^2^ and 91,347,736 respectively bounded on the north by Sikkim and Bhutan, on the east by Assam and Bangladesh, on the south by the Bay of Bengal and on the west by Orissa, Bihar, Jharkhand and Nepal [22]. In 1973 JE outbreak was first recorded in the rural districts of Burdwan and Bankura in the state of WB where 700 cases and 300 deaths were reported [23,24]. Thereafter, several JE outbreaks took place in the state [25-27]. As per published literature, the State Health Department, Govt. of WB has conducted vaccination programme against JE in different rural districts of WB [28]. But still sporadic JE/AES cases and deaths are being reported every year from the state [29]. The people of the state dependent on cultivation work in the water-logged paddy fields which serve as congenial home for mosquito breeding [22]. Moreover, to raise their economic status they usually take up piggery and mini-poultry in their own hut bringing animals in their close association. In addition, environmental factors of this state also favor JEV transmission [22]. Moreover, the reports of JE incidences or endemicity of JE in the state might be the indications of partial vaccination although the emergence of mutated/new strain of JEV could not be excluded.

The present study was carried out to characterize the genetic variation in the E protein of JEV circulating in the WB state and to observe the difference and/or crucial amino acid changes in E protein of the WB isolates in comparison with SA14-14-2 live attenuated vaccine strain in order to enable us to understand the variations in antigenic and neutralizing properties of different JEV WB isolates, followed by analyzing the molecular impact of amino acid substitutions on envelope protein structure of those isolates in relation to disease severity.

## Methods

### Case enrollment and sample collection

Clinically diagnosed 98 AES cases (28 from Malda, 15 from Hooghly, 25 from Midnapore, 11 from Birbhum, 10 from Howrah and 9 from South 24 Parganas) of all age groups having high grade fever for ≤15 days with the symptoms viz. headache, vomiting, unconsciousness/coma, convulsion/seizure, abnormal movements, delirium, altered sensorium, neck rigidity, presence of kernig’s sign etc. were admitted in abovenoted 6 district government hospitals in the state of WB from August to December both in 2011 and 2012. The study was duly approved by the joint ethical committee of ICMR (Indian Council of Medical Research) virus unit and NICED (National Institute of Cholera and Enteric Diseases), Kolkata, India. Informed consents were obtained in prescribed proforma from the patients or legal guardians or relatives of the patients before the collection of the samples. A total of 98 [62 serum and 36 cerebrospinal fluid (CSF)] samples were collected and/or referred from the above said clinically diagnosed AES case-patients to ICMR Virus Unit, Kolkata, maintaining the cold chain, for the detection of JEV infection. Cerebral malaria and bacteriological etiology were ruled out on the basis of clinical observations by the physicians of the hospitals concerned. All sera and CSF samples were stored in aliquots at −80°C until use.

### IgM-capture ELISA

All the samples were tested to detect the presence of JEV specific immunoglobulin M (IgM) antibody using JEV IgM-capture ELISA test kit (supplied by National Institute of Virology, Pune, India) based on the manufacturers’ protocol. Optical density (O.D) was measured at 450 nm using an ELISA reader (Titertek Multiskan Plus, Lab systems Finland, Type-314).

### Isolation of virus from samples

Only 24 JEV IgM negative samples with a history of ≤3 days’ illness were screened and 200 μl of each of them was inoculated to C6/36 cell line for virus isolation as previously described [28]. The tissue culture fluids were collected from the samples producing prominent cytopathic effect (CPE), and centrifuged at 1000 × g for 5 minutes and the supernatants of tissue culture fluids (STF) were kept in aliquots at −80°c till the isolation/extraction of viral RNA, followed by E gene amplification through RT-PCR test.

### RNA extraction, E gene amplification and nucleotide sequencing

Viral RNA was extracted from 140 μl of STF utilizing QIAamp RNA viral kit (Qiagen, GmbH, Hilden, Germany), following the manufacturer’s instructions. Extracted/purified RNA was applied as template for cDNA synthesis using avian myeloblastosis virus (AMV) reverse transcriptase (RT) and the JEV E gene specific reverse primer. In brief, the target RNA was converted to cDNA in 25 μl mixture volumes containing the following components: 11.76 μl of dH_2_O (MiliQ grade), 5 μl of 5× AMV RT buffer, 1.6 μl of 10 mM deoxynucleotide triphosphates (dNTP_S_) (Invitrogen, USA), 0.1 μl of 0.1 M dithiothreitol (DTT) (Promega, USA), 1.5 μl of 10 μM JEV E gene specific reverse primer [28], 0.04 μl of 10 U/μl AMV RT (Promega, USA) and 5 μl of RNA (50 pg to 1 μg). The reaction mixture was allowed to cDNA synthesis for 1 h at 42°c. The cDNA was subsequently used for polymerase chain reaction (PCR) amplification in the total of 20 μl reaction mixture that was prepared using 9.3 μl of dH_2_O (MiliQ grade), 2 μl of 10× DreamTaq DNA polymerase buffer, 0.5 μl of 10 mM deoxynucleotide triphosphates (dNTP_S_) (Invitrogen, USA), 1.5 μl of 10 μM JEV E gene specific primer pairs [28], 0.2 μl of 5U/μl DreamTaq DNA polymerase (Fermentas Inc., USA) and 5 μl of cDNA. PCR reaction conditions were as follows: initial denaturation at 94°c for 5 min, followed by 35 cycles of denaturation (at 94°c for 30 s), primer annealing (at 67°c for30 s), and primer extension (at 72°c for 1 min 30 s). Then a final extension step was carried out at 72°c for 5 min. The PCR products were separated by electrophoresis on 1% agarose gel, stained with ethidium bromide.

RT-PCR amplicons were purified with the help of the Qiagen gel extraction kit (Qiagen, GmbH, Hilden, Germany), according to the manufacturer’s protocol and subjected to direct sequencing using the BigDye Terminator Cycle Sequencing Ready Reaction Kit (Applied Biosystems, Foster City, CA, USA), in accordance with manufacturer’s specifications. The products were purified by ethanol precipitation and were analyzed by an automated DNA sequencer, 3130XL Genetic Analyzer (PE Applied Biosystems, Foster city, CA, USA). The freely available Finch TV software (http://www.geospiza.com) was used to edit and correct the 1,500 nucleotides which generated complete E gene sequences of JEV isolates. Those sequences were subjected to BLAST search.

### Multiple sequence alignment (MSA) and phylogenetic analysis

The E gene sequences of JEV strains/isolates used in MSA and phylogenetic analysis in this study were listed in Additional file 1. MSA and phylogenetic analysis were performed by CLUSTALW (http://www.ebi.ac.uk/Tools/clustalw2/index.html) and MEGA version 5.0 software (http://www.megasofteware. net). The phylogenetic tree was constructed by the neighbor-joining method, tested with Kimura 2-parameter model and evaluated by 1000 bootstrap pseudo replicates. The strain MVEV-1-51 was used as an out group for generating the rooted tree. Moreover, the structure-based MSA of JEV isolates with respect to SA14-14-2 vaccine strain were analyzed by an online server named Porter (http://www.distill.ucd.ie/porter/) to confirm the alteration of E protein secondary structure regarding disease severity.

### Mapping of amino acid substitutions

Amino acid sequences were deduced from nucleotide sequences of the JEV isolates by Transeq (http://www.ebi.ac.uk/Tools/emboss/transeq/). The comparisons were made to identify the JEV isolates-specific amino acid substitutions in relation to JEV SA14-14-2 vaccine strain and were subsequently mapped onto the predicted 3-dimensional structures of isolate-specific E protein, as modelled based on predicted similarities to the homologous (95.8% - 97.8% identity) E protein of JEV SA14-14-2 vaccine strain [PDB:3P54] using ESyPred3D web server (http://www.fundp.ac.be/sciences/biologie/urbm/bioinfo/esypred/). Finally, the predicted JEV isolate-specific E protein structures were viewed and manipulated with the help of PyMol software (http://www.pymol.org/).

### T-cell epitope prediction

T-cell epitopes of the JEV isolate-specific structural E proteins were predicted using the EpiJen online server (http://www.ddg-pharmfac.net/epijen/) with respect to JEV SA14-14-2 vaccine strain. The isolate-specific amino acid sequences of E protein were put to use as input to EpiJen including JEV SA14-14-2 vaccine strain and the server was run in the default mode. The appropriate proteasomal and tap cut-off values were selected during epitope predictions for the 18 different HLA alleles.

## Results

### Serology, virus isolation and RT-PCR

Out of 98 samples, only 39 (39.7%) were reactive to JEV specific IgM antibody, of which 24 (61.5%) and 15 (38.4%) samples were CSF and serum respectively. Of the remaining 59, only 24 JEV IgM negative samples having the history of ≤3 days of febrile illness were screened and subsequently subjected to tissue culture for virus isolation. This resulted in 11 samples producing prominent CPE (characterized by the roundening of cells, increased granularity and vacuolation, followed by cell death and disruption of the monolayer by detachment of the dead cells) of which only 7 (29.1%) samples consisting of 5 (71.4%) from CSF and 2 (28.5%) from serum were determined to be JE with positive findings of viral RNA by RT-PCR test.

The basic characteristics of the patients from whom the samples were collected and selected/subjected to tissue culture for virus isolation, were presented in Table 1. The patients from the districts of Midnapore, Malda, Birbhum, Howrah and South 24 Parganas in WB state were all pediatric-adolescents, with an average age of 8–13 years whereas the patients belonging to Hooghly district were all adults, with an average age of 47 years. The summarized data of the patients from whom the viruses were isolated in the study was depicted in Table 2.

**Table 1:** Basic characteristics of the patients whose samples were screened for virus isolation in this study

**Study area**	**No. of patients**	**Sex**		**Average age**
		**Male**	**Female**	**(Years) (Range)**
Midnapore, West Bengal	7	4	3	8 (3–13)
Hooghly, West Bengal	3	2	1	47 (39–55)
South 24 Parganas, West Bengal	3	2	1	12 (3–17)
Howrah, West Bengal	1	0	1	13 -
Malda, West Bengal	5	3	2	12 (7–16)
Birbhum, West Bengal	5	3	2	10 (5–16)

**Table 2:** Short history of the patients from whom JEVs were isolated

**No. of patients**	**Residence**	**JE vaccination history**	**Date of onset**	**Samples collected on**	**Sample type**	**Clinical outcome**	**Results of**		**Isolates (GenBank accession no.)**	**Genotype**
							**ELISA (IgM) RT-PCR**			
Patient 1	Midnapore	Yes	19/08/2011	21/08/2011	CSF	Moderate	Negative	Positive	IND/11/WB/JEV45 (KC526872)	Genotype I
Patient 2	Midnapore	No	23/09/2011	25/09/2011	CSF	Severe	Negative	Positive	IND/11/WB/JEV46 (KC526869)	Genotype III
Patient 3	Hooghly	Unknown	27/09/2011	28/09/2011	CSF	Expired	Negative	Positive	IND/11/WB/JEV47 (KC526870)	Genotype III
Patient 4	South 24 Parganas	No	11/10/2011	13/10/2011	CSF	Severe	Negative	Positive	IND/11/WB/JEV48 (KC802020)	Genotype III
Patient 5	Howrah	No	20/08/2011	22/08/2011	Serum	Severe	Negative	Positive	IND/11/WB/JEV49 (KC802021)	Genotype III
Patient 6	Malda	No	25/09/2012	28/09/2012	CSF	Mild	Negative	Positive	IND/12/WB/JEV50 (KC526871)	Genotype III
Patient 7	Birbhum	No	22/09/2012	25/09/2012	Serum	Mild	Negative	Positive	IND/12/WB/JEV51 (KC802022)	Genotype III

However, we have a total of 46 (39 IgM + 7 RT-PCR positive) JE cases representing 46.9% of 98 cases. Out of 46 cases, 33 (27 IgM + 6 RT-PCR positive) were pediatric-adolescents (71.7%) and remaining 13 (12 IgM + 1 RT-PCR positive) were found to be adult cases (28.2%). Moreover, the occurrence of JEV infection was recorded for the months of August to October in 2011 and 2012, with the maximum number of cases (71.4%) observed in September in 2 consecutive years.

### Molecular phylogeny and sequence analysis of isolates

**Figure 1:**
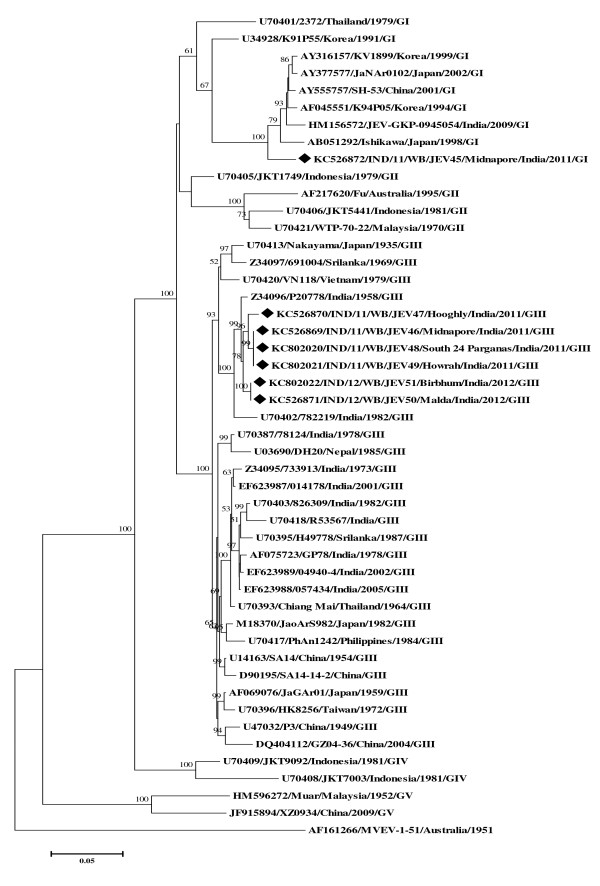
**Phylogenetic relationship among JEV isolates from WB, India.** The Neighbor-Joining (NJ) Phylogenetic tree, tested with Kimura 2-parameter model was generated by MEGA5, using the complete E gene nucleotide sequences of 4 JEV isolates from hospitalized AES case-patients in West Bengal during 2011–12, with reference to other 40 wild type JEV strains from worldwide. The strain MVEV-1-51 was used as an out group for generating the rooted tree. The ≥50% bootstrap support values (1000 pseudo replicates) were shown in corresponding nodes. Horizontal branch lengths are proportional to genetic distance and vertical branch lengths have no significance. Each taxon is named systematically by mentioning the accession number, strain/isolate name, country of origin, year of isolation and genotype. The isolates’ sequences used in this study were indicated by ‘♦’ mark. Scale bar indicates nucleotide substitutions per site.

The Figure 1 represented the phylogenetic tree derived from 7 E gene sequences of JEV isolates along with 40 wild-type JEV strains, including 11 from India and 29 from worldwide (Additional file 1). Phylogram showed 6 E gene sequences of the isolates [GenBank:KC526869-KC526871 and KC802020-KC802022] belonging to GIII and comprising 98%-100% nucleotide similarity with each other and 93%-98% nucleotide similarity with other Indian GIII strains, having the highest similarity (97%-98%) with Indian P20778 strain [GenBank:Z34096] and those GIII E gene sequences also amounted to 95%-96% nucleotide similarity with GIII SA14-14-2 vaccine strain [GenBank:D90195]. On the other hand, 1 E gene sequence of another isolate [GenBank:KC526872] belonging to GI that was most similar (96%) with Japanese GI strain Ishikawa [GenBank:AB051292], followed by 94% nucleotide similarity with other Indian GI isolate JEV-GKP-0945054 [GenBank:HM156572] and also having 87% nucleotide similarity with above said vaccine strain. Above all, the E gene nucleotide sequences of GIII isolates comprised 89%-90% nucleotide identity with GI isolate.

**Figure 2:**
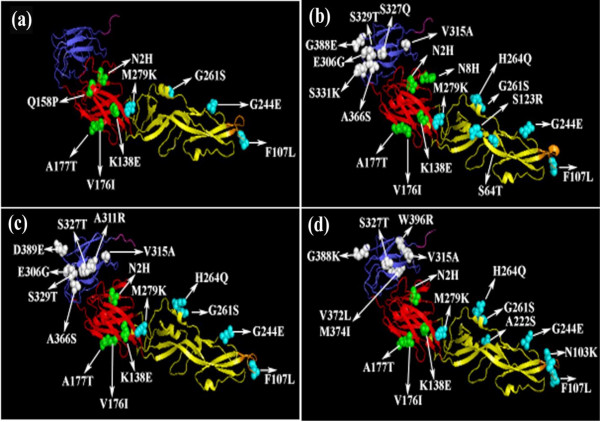
**Structural locations of JEV isolate-specific amino acid variations in E protein.** Predicted 3-dimensional structures of JEV isolates-specific E protein [2 **(a)** - 2 **(d)** for IND/12/WB/JEV50, IND/11/WB/JEV47, IND/11/WB/JEV46 and IND/11/WB/JEV45 respectively] derived from the crystal structure of E protein of JEV SA14-14-2 vaccine strain, showing amino acid substitutions in different domains i.e. domain I (red), domain II including orange colored fusion loop (yellow) and domain III (blue) as designated in earlier studies [32,33].

The isolates IND/11/WB/JEV48 [GenBank:KC802020] and IND/11/WB/JEV49 [GenBank:KC802021] were identical (having 100% nucleotide similarity) with isolate IND/11/WB/JEV46 [GenBank:KC526869] and all of them showed same clinical outcome in the patient 4, 5 and 2 (Table 2). Moreover, 100% nucleotide similarity was also observed in between the isolate IND/12/WB/JEV51 [GenBank:KC802022] and IND/12/WB/JEV50 [GenBank:KC526871] and both of them were associated with mild JE in the patient 7 and 6 (Table 2). Since, the isolates IND/11/WB/JEV48, IND/11/WB/JEV49 and IND/12/WB/JEV51 were 100% identical with isolates IND/11/WB/JEV46 and IND/12/WB/JEV50, molecular modelling and T-cell epitope prediction data of the isolates IND/11/WB/JEV48, IND/11/WB/JEV49 and IND/12/WB/JEV51 were not shown in Figure 2 and/or in Tables 3 and 4 respectively except the other isolates.

**Table 3:** Comparison of E protein based amino acid substitutions identified in WB isolates with respect to wild type SA14-14-2 vaccine stain of JEV

**Sl. no.**	**Identified E protein domains**	**Positions wise amino acid substitutions**	**Types of amino acid substitutions**^**a**^	**Wild type vaccine strain**	**WB Isolates**			
				**SA14-14-2**	**IND/12/WB/JEV50**	**IND/11/WB/JEV47**	**IND/11/WB/JEV46**	**IND/11/WB/JEV45**
1	Domain I	N2H	NC	N	H	H	H	H
2	Domain I	N8H	NC	N	.	H	.	.
3	Domain I	K138E	NC	K	E	E	E	E
4	Domain I	Q158P	C	Q	P	.	.	.
5	Domain I	V176I	C	V	I	I	I	I
6	Domain I	A177T	NC	A	T	T	T	T
7	Domain II	S64T	C	S	.	T	.	.
8	Domain II	N103K	NC	N	.	.	.	K
9	Domain II	F107L	NC	F	L	L	L	L
10	Domain II	S123R	NC	S	.	R	.	.
11	Domain II	A222S	NC	A	.	.	.	S
12	Domain II	G244E	NC	G	E	E	E	E
13	Domain II	G261S	NC	G	S	S	S	S
14	Domain II	H264Q	NC	H	.	Q	Q	Q
15	Domain II	M279K	NC	M	K	K	K	K
16	Domain III	E306G	NC	E	.	G	G	.
17	Domain III	A311R	NC	A	.	.	R	.
18	Domain III	V315A	C	V	.	A	A	A
19	Domain III	S327Q/T	C	S	.	Q	T	T
20	Domain III	S329T	C	S	.	T	T	.
21	Domain III	S331K	NC	S	.	K	.	.
22	Domain III	A366S	NC	A	.	S	S	.
23	Domain III	V372L	C	V	.	.	.	L
24	Domain III	M374I	C	M	.	.	.	I
25	Domain III	G388E	NC	G	.	E	.	K
26	Domain III	D389E	C	D	.	.	E	.
27	Domain III	W396R	NC	W	.	.	.	R
28	Stem	R416K	C	R	K	K	.	.
29	Stem	G432R	NC	G	.	R	.	.
30	Stem	R439K	C	R	.	K	K	K
31	Transmembrane	L463R	NC	L	.	.	R	.
32	Transmembrane	L467E	NC	L	.	E	.	.
33	Transmembrane	A481D	NC	A	.	D	.	.

Different amino acid substitutional positions were categorized into domains based on earlier reports [32,33].

Identical amino acids in WB isolates with respect to wild type SA14-14-2 vaccine stain of JEV were indicated by “.”.

^a^Amino acid substitutions were designated as C for conservative and NC for non-conservative.

**Table 4:** Identification of E protein based amino acid substitutions in JEV WB isolates with potential T-cell epitopes

**Sl. no.**	**Identified E protein domains**	**Amino acid substitutions with positions**	**Associated HLA alleles**^**#**^	**WB Isolates with amino acid substitutions**			
				**IND/12/WB/JEV50**	**IND/11/WB/JEV47**	**IND/11/WB/JEV46**	**IND/11/WB/JEV45**
1	Domain I	N2H	-	Y	Y	Y	Y
2	Domain I	N8H	-	-	Y	-	-
3	Domain I	K138E	HLA-A*24	Y	Y	Y	Y
4	Domain I	Q158P	HLA-A*1101, HLA-A*24	Y	-	-	-
5	Domain I	V176I	HLA-A*0101, HLA-A*0301,	Y	Y	Y	Y
			HLA-A*1101, HLA-A*6801,				
			HLA-A*6802, HLA-B*07, HLA-B*51				
6	Domain I	A177T	HLA-A*0101, HLA-A*0301,	Y	Y	Y	Y
			HLA-A*1101, HLA-A*6801,				
			HLA-B*07, HLA-B*51				
7	Domain II	S64T	HLA-A*0202, HLA-A*0203,	-	Y	-	-
			HLA-A*0206, HLA-A*1101,				
			HLA-A*24, HLA-A*6802,HLA-B*27				
8	Domain II	N103K	HLA-A*24	-	-	-	Y
9	Domain II	F107L	HLA-A*1101, HLA-A*24, HLA-B*51	Y	Y	Y	Y
10	Domain II	S123R	HLA-A*0301, HLA-A*1101,	-	Y	-	-
			HLA-A*24, HLA-A*6801, HLA-B*27				
11	Domain II	A222S	HLA-B*40, HLA-B*44	-	-	-	Y
12	Domain II	G244E	HLA-A*0101, HLA-A*0201,	Y	Y	Y	Y
			HLA-A*0301				
13	Domain II	G261S	-	Y	Y	Y	Y
14	Domain II	H264Q	HLA-A*0202, HLA-A*0206, HLA-B*51	-	Y	Y	Y
15	Domain II	M279K	HLA-A*0301, HLA-A*1101,	Y	Y	Y	Y
			HLA-A*24, HLA-A*6802, HLA-B*07, HLA-				
			B*40, HLA-B*44				
16	Domain III	E306G	HLA-A*0301, HLA-A*1101, HLA-A*24	-	Y	Y	-
17	Domain III	A311R	HLA-A*0301, HLA-A*1101, HLA-B*27	-	-	Y	-
18	Domain III	V315A	-	-	Y	Y	Y
19	Domain III	S327Q	HLA-A*0101, HLA-A*0301,	-	Y	-	-
			HLA-A*1101, HLA-A*24				
		S327T	HLA-A*0101, HLA-A*0301,	-	-	Y	Y
			HLA-A*1101, HLA-A*24				
20	Domain III	S329T	HLA-A*0301, HLA-A*1101,	-	Y	Y	-
			HLA-A*24, HLA-B*51				
21	Domain III	S331K	HLA-A*0301, HLA-A*1101,	-	Y	-	-
			HLA-A*24, HLA-B*51				
22	Domain III	A366S	HLA-A*0101, HLA-A*0201,	-	Y	Y	-
			HLA-A*0202, HLA-A*0206,				
			HLA-A*0301, HLA-A*1101,				
			HLA-B*40, HLA-B*44				
23	Domain III	V372L	-	-	-	-	Y
24	Domain III	M374I	-	-	-	-	Y
25	Domain III	G388E	HLA-A*0301, HLA-A*6802,	-	Y	-	-
			HLA-B*27				
		G388K	HLA-A*0301, HLA-A*1101,	-	-	-	Y
			HLA-A*6802, HLA-B*07, HLA-B*27				
26	Domain III	D389E	HLA-A*0301, HLA-A*1101, HLA-A*6802, HLA-B*27, HLA-B*40, HLA-B*44	-	-	Y	-
27	Domain III	W396R	-	-	-	-	Y
28	Stem	R416K	-	Y	Y	-	-
29	Stem	G432R	-	-	Y	-	-
30	Stem	R439K	-	-	Y	Y	Y
31	Transmembrane	L463R	-	-	-	Y	-
32	Transmembrane	L467E	-	-	Y	-	-
33	Transmembrane	A481D	-	-	Y	-	-

^#^ Predicted peptide-epitopes with scores were given as Additional file 3 and Additional file 4. Presence of amino acid substitutions in WB isolates associated with HLA alleles was indicated by “Y”.

Among several nucleotide changes, some were non-synonymous (data not shown) resulting in amino acid substitutions in JEV isolate-specific E protein in comparison with above said vaccine strain (Table 3). Moreover, the 8 amino acid substitutions (N2H, F107L, K138E, V176I, A177T, G244E, G261S and M279K) were common to all isolates whereas isolate-specific unique amino acid substitutions were also shown by a structure-based MSA of JEV isolates and SA14-14-2 vaccine strain [Additional file 2 (b)] which might confirm the alteration of E protein secondary structure with respect to disease severity.

### Molecular modelling of isolates-specific E protein

Homology modelling of JEV isolates-specific 3-dimensional structures of E protein mapped with amino acid substitutions in relation to SA14-14-2 vaccine strain have been shown in Figure 2 (a)–(d). The amino acid substitutions were pointed out to areas with major secondary structures likely to affect the protein conformation regarding disease severity.

### T-cell epitope prediction

Amino acid substitutions in E proteins of JEV isolates with potential T-cell epitopes were predicted in Table 4 and the substitutions in peptide-epitope sequences of the isolates with greater/lower IC50 values/scores were also given in Additional file 3 and Additional file 4 (actual Amino acid residues in peptide-epitope sequences of vaccine strain SA14-14-2 with IC50 values/scores were not shown). As a result, it is interesting to note that majority of the identified mutations falling within predicted T-cell peptide-epitopes might influence/alter immunogenicity/antigenicity in relation to said vaccine strain. While ranking the epitopes for mutation mapping, epitopes with calculated IC50 values/scores above 100 were not included since such peptide-epitopes were unlikely to yield significant immunogenicity.

## Discussion

The virulence of JEV etiology is of grave public health concern in the state of WB. The present study reveals that 39/98 (39.7%) and 7/24 (29.1%) samples were positive to JE by IgM-capture ELISA and RT-PCR method respectively. This observation proves that JE occurs recently and the ELISA negative acute samples with febrile illness for ≤ 3 days should be subjected to RT-PCR test to confirm the total number of JE cases.

Pediatric-adolescents (71.7%) were higher with JE cases than adults (28.2%) as because pediatrics were infected possibly due to lack of immunity and adolescents were directly exposed to the mosquito vector (Culex sp.) bite, as they usually took active part in cultivation in water-logged crop fields where vectors usually breed. In contrast, low numbers of JE cases in adults were possibly due to the development of immunity by sub-clinical infections in natural time scale. In the present study, the maximum number of JE cases was found to occur in the monsoon period i.e. in the month of September when the Culex mosquitoes breed in the paddy fields covered with stagnant rain water.

However, we proposed that 52 [(59–24) + (24–7)] samples with a history of 1–15 days’ illness were true JE negative possibly due to either mishandling of samples which damaged the IgM antibody/the viral titre or the presence of another etiology responsible for AES.

In molecular phylogeny, JEV GIII isolates i.e. IND/11/WB/JEV46 [GenBank:KC526869], IND/11/WB/JEV47 [GenBank:KC526870], IND/11/WB/JEV48 [GenBank:KC802020], IND/11/WB/JEV49 [GenBank:KC802021], IND/12/WB/JEV50 [GenBank:KC526871] and IND/12/WB/JEV51 [GenBank:KC802022] showed closer clustering, even though all of 6 isolates were from a geographically distant location or different districts of the state (Table 2, Figure 1). This indicated similarities among the isolates circulating in the 2 consecutive years 2011, 2012. Moreover, based on the present study, we stated that there had been simultaneous circulation of both JEV GI [GenBank:KC526872] and GIII [GenBank:KC526869] in the district of Midnapore, WB in 2011 corroborating with our earlier findings [21]. It would be interesting to determine whether GI has become the predominant genotype or co-circulate with GIII in WB, India; additional JEV isolates from human clinical samples will be required to confirm the findings. Our study also reveals that the JEV GI [GenBank:KC526872] was isolated from a 9-year old boy-patient who had already been immunized with live attenuated JE vaccine derived from GIII strain SA14-14-2 and the boy was found to have clinically developed moderate JE with high fever including unconsciousness, neck rigidity and convulsion. In this connection, the efficacy of the vaccine to protect against GI isolate of JEV needs careful evaluation. In addition, we have identified the samples from 3 adult patients (average age of 47 years) with AES belonging to district Hooghly, WB were subjected to tissue culture for JEV isolation (Table 1) and out of these 3 samples, only 1 was confirmed as JE positive by RT-PCR (Table 2). This observation suggested that adults in this district were at higher risk for JE. Therefore, the state health department, government of WB should take initiative about JE vaccination to this population to overcome the disease burden.

The 500 amino acids long JEV E protein is encoded by E gene of 1500 nucleotides in length. The E protein of flaviviruses, including JEV, plays an important role in immunogenicity, tissue tropism, cell fusion and infection, and virus maturation [30]. The predicted 3-dimensional structures of isolate-specific E protein were modelled onto the crystal structure of JEV SA14-14-2 vaccine strain E protein [31] and composed of separate structural domains [designated as domain I (DI), domain II (DII) and domain III (DIII)] including stem (ST) and transmembrane (TM) region like other flaviviruses [32,33]. DI, which is referred to as the central domain, consists of 127 residues (1–51, 135–193 and 283–299); DII, which is called as dimerization domain, consists of 172 residues (52–134 and 194–282); the most antigenic part of E protein is DIII, a continuous stretch of 100 residues (300–399) that has been implicated in receptor binding; a stretch of 52 residues (400–451) is considered as ST region of E protein not simply to support the protein, but rather, it plays a key role in viral entry and maintenance of the protein complexes on the virion surface followed by TM, a 49 residues (452–500) long region playing a major role in the processing, sub-cellular localization and assembly of E protein [Additional file 2 (a)].

In the present study, in order to identify the possible correlation of clinical severity (i.e. neurovirulence/neuroinvasiveness) and altered immunogenicity of JEV infection in WB with isolate-specific amino acid changes of E protein, we analyzed E protein amino acid changes of the viral isolates with respect to SA14-14-2 vaccine strain. Our study showed greater amino acid changes in the JEV isolates circulating in WB than that of SA14-14-2 (Table 3). It was found that 8 critical amino acid changes at E107 (F→L), E138 (K→E), E176 (V→I), E177 (A→T), E264 (H→Q), E279 (M→K), E315 (V→A) and E439 (R→K) (Table 3) of 3 isolates i.e. IND/11/WB/JEV45-IND/11/WB/JEV47 (except the isolate IND/12/WB/JEV50) display neurovirulence in the patient 1–3 respectively (Table 2). That type of severity due to JEV infection worldwide or in earlier reports [14,34-37] has been linked to the amino acid alterations in the virus. Moreover, the above said eight critical amino acid changes including E123 (S→R) and E306 (E→G) in the isolate IND/11/WB/JEV47 and only E306 (E→G) in the isolate IND/11/WB/JEV46 (Table 3) might introduce much more neurovirulence/neuroinvasiveness corroborating with previous findings [38,39] and the increased virulence of the isolates is likely to cause death and severe JE/AES in the patient 3 and 2 respectively (Table 2). In contrast, although the isolate IND/12/WB/JEV50 showed critical amino acid changes, it did not include H→Q at E264, V→A at E315 and R→K at E439 which were found to be closely associated with neurovirulence (Table 3). Therefore, it might result in low neurovirulence in the patient 4 who developed mild JE/AES (Table 2). Moreover, regarding the low neurovirulence property of the isolate IND/12/WB/JEV50, it was speculated that a novel conservative amino acid substitution i.e. Q158P was mapped onto an α-helix of glycosylated E_0_F_0_ loop [31,40] in DI of that isolate’s E protein [Figure 2 (a)] and was found to have potential to break (as because the proline is a helix breaker) the secondary structural elements surrounding that position and might also affect the N^154^ glycosylation site [41] of E protein of the isolate [Additional file 2 (b)], being less severe for the patient 4.

The crystal structure of E protein of WNV, a Flavivirus closely related to JEV [42], illustrated that the finger like projection of a stretch of 13 residues (98–110) long fusion loop (FL) is also present in the DII of JEV E protein, essential for viral infectivity, membrane fusion and is recognized as neutralizing epitope [31,43]. In the study, Hopp and Woods hydrophilicity prediction [44] revealed that the non-conservative substitution of basic lysine (K) for polar asparagine (N) resulted in decreased (IC50 value/score) hydrophilicity of ^98^DRGWG**K**GCGLFKG^110^ peptide sequence of the isolate IND/11/WB/JEV45 E protein [Table 4, Additional file 2(b) and Additional file 3]. This in turn, indicates altered antigenicity of the isolate in comparison with SA14-14-2 vaccine strain. Moreover, we found that another non-conservative amino acid change i.e. E107 (F→L) at the tip of FL region of E protein of all JEV isolates circulating in WB might lead to escape from antibody neutralization or neutralizing epitope with decreased/increased IC50 value/score in relation to said vaccine strain [Table 4, Additional file 2(b), Additional file 3 and Additional file 4]. This finding was supported by previous studies [31,45].

It is noteworthy that the amino acid substitutions at E64 (S→T), E244 (G→E), E306 (E→G) and E311 (A→R) in the DII and DIII of JEV isolate-specific E protein with respect to said vaccine strain [Table 4, Additional file 2(b), Additional file 3 and Additional file 4] might largely change immunogenicity (with decreased/increased IC50 value/score) of the isolates corroborating with earlier reports [33,40,46-49]. According to earlier workers [32,40,50], the surface exposed BC loop (E229-E333), DE loop (E365-368) and the putative receptor binding RGD motif (E387-E389), adjacent to FG loop (E389-E391) region were recognized as most antigenic part of DIII region of WNV E protein (closely related to JEV). In the present study, some amino acid substitutions (i.e. S327Q/T, S329T, S331K, A366S, G388E/K and D389E) were observed within and/or adjacent to BC loop, DE loop and RGD associated FG loop region of the JEV isolates’ (IND/11/WB/JEV45-IND/11/WB/JEV47) E protein (Table 3). In Hopp and Woods hydrophilicity prediction [44], both conservative and non-conservative substitutions of S327Q/T, S329T, S331K (polar to charged), A366S (non-polar to polar) and G388E/K (non-polar to charged) resulting in increased hydrophilicity of the ^327^**Q**Y**T**G**K**DG^333^ / ^327^**T**Y**T**GSDG^333^ / ^327^**T**YSGSDG^333^, ^365^S**S**NV^368^ and ^387^R**E**DKQ^391^ / ^387^R**K**DKQ^391^ peptide sequences of above mentioned JEV isolates in relation to SA14-14-2 vaccine strain might lead to escape from antibody neutralization or neutralizing epitope due to increased/decreased IC50 value/score [Table 4, Additional file 2(b), Additional file 3 and Additional file 4] whereas another conservative amino acid change at E389 (D→E) in the BC loop region of E protein of the isolate IND/11/WB/JEV45 retained the same hydrophilicity of the ^387^RG**E**KQ^391^ peptide sequence in comparison with said vaccine strain.

Several mutations in the structural E protein gene region were seen to fall within the predicted T-cell epitopes (Table 4) as a result of analysis of the changes that might affect the immunogenicity of these isolates. Involvement of epitopes presented by major HLA alleles, such as HLA-A*02 and HLA-A*11 were shown by the present study. Moreover, HLA-A*11, one of the main HLA types, is found to be present in population across the world particularly with its high prevalence in South East Asia [51]. When the variations in these epitopes take place, they may have the potential to affect the cell-mediated immune response against JEV infection.

It is worthy to mention that substitutions of non-polar to charged amino acid residues at E432 (G→R), E463 (L→R), E467 (L→E) and E481 (A→D) in the isolate-specific ST and TM region of E protein (Table 3) might facilitate the processing, sub-cellular localization and assembly of E protein of the isolates, which was proved by earlier study [52]. Interestingly, the present JEV GI isolate IND/11/WB/JEV45 had a non-conservative amino acid substitution at E222 (A→S) corresponding to DII region of its E protein [Table 3, Figure 2 (d), Additional file 2 (b)] which was also observed in the previous study [53]. Such type of mutation might influence/facilitate vector competency and viral infectivity. This was similar to the recent findings [54]. All WB isolates showed amino acid mutations at E2 (Asn to His) and E261 (Gly to Ser) and the isolate-specific mutations i.e. N8H of IND/11/WB/JEV47; R416K of IND/11/WB/JEV47 and IND/12/WB/JEV50 and V372L, M374I and W396R of IND/11/WB/JEV45 were also observed in the study (Table 3), but the biological significance of those mutations were unknown.

## Conclusions

In summary, our study reveals that JEV GI and GIII co-circulate with genetic changes in the state of WB, India during 2011–12. The JEV GI isolate IND/11/WB/JEV45 [GenBank:KC526872] from a 9-year old boy-patient who had been vaccinated with the live attenuated JE vaccine derived from JEV GIII SA14-14-2 strain but developed moderate JE/AES as because the GI isolate differed from the JEV GIII vaccine strain by means of some amino acid substitutions which were responsible for both neurovirulence and alteration of immunogenicity/antigenicity. Therefore, the vaccine efficacy to protect against JEV GI may demand careful evaluation. However, using a bioinformatics approach we point out that this study is a localized example of molecular evolution of E protein gene of JEV isolates in nature, which is likely to affect the disease profile. Further, functional studies on these mutant isolates would help us to understand the correlation of the genetic changes with respect to JEV virulence and pathogenesis.

### Ethical approval

The study was duly approved by the joint ethical committee of ICMR (Indian Council of Medical Research) virus unit and NICED (National Institute of Cholera and Enteric Diseases), Kolkata, India.

## Competing interests

The authors declare that they have no competing interests.

## Authors’ contributions

AS conceived the study, the design, and drafted the manuscript. AS carried out serology and molecular work. AS, AB, BKP participated in immunoinformatics analysis. AS, AB, BKP, SKM and SC contributed to the data analysis and data interpretation. All authors read and approved the final manuscript.

## Pre-publication history

The pre-publication history for this paper can be accessed here:

http://www.biomedcentral.com/1471-2334/13/368/prepub

## Supplementary Material

Additional file 1 **Sources of the JEV strains/isolates used in the phylogenetic analysis in this study.** All strains/isolates are JEV, except Murray Valley encephalitis virus strain (MVE-1-51) used as an out group for phylogenetic analysis in this study. */** Isolates were 100% identical with each other. Click here for file

Additional file 2 **(a) Schematic structural representation of E protein of JEV isolates.** Here, DI, DII (FL), DIII, ST and TM were symbolized for domain I (red), domain II with orange colored fusion loop (yellow), domain III (blue), stem (magenta) and transmembrane (gray) region respectively including the span/stretch of respective domain/region with above shown numbering, according to previous reports [32, 33]. **(b)** A comparison of structure-based multiple sequence alignment of JEV isolates-specific E protein with respect to SA14-14-2 vaccine strain. Domains/regions were colored as Figure 2 (a). Secondary structural elements (described in terms of E-extended strand/β-sheet, H-α-helix and C-coil/turn/bend) were shown below each sequence alignment. Underlined bold faces amino acids represent the JEV isolates-specific amino acid substitutions in E protein. Amino acid substitutions leading to escape from antibody neutralization or neutralizing epitopes or related to neuorovirulence/neuroinvasiveness or both found in the JEV isolates with respect to SA14-14-2 vaccine strain were represented by shaded dot, dot and bold asterisk (*) respectively. One amino acid substitution at E158 (Q→P) in the isolate IND/12/WB/JEV50 resulting in secondary structural changes marked as green shaded bold faces. Click here for file

Additional file 3 **Amino acid substitutions in E protein epitopes of JEV WB isolates associated with HLA-A alleles as predicted by EpiJen serve.** The values given within the bracket indicates the 50% inhibitory concentration (IC50) of the peptide, a measure of the binding affinity. An IC50 value < 50 is considered as a good affinity. Amino acid substitutions in the predicted epitopes of E protein are marked as bold. Click here for file

Additional file 4 **Amino acid substitutions in E protein epitopes of JEV WB isolates associated with HLA-B alleles as predicted by EpiJen server.** The values given within the bracket indicates the 50% inhibitory concentration (IC50) of the peptide, a measure of the binding affinity. An IC50 value < 50 is considered as a good affinity. Amino acid substitutions in the predicted epitopes of E protein are marked as bold. Click here for file
