# Supplementary material for: Envelope protein gene based molecular characterization of Japanese encephalitis virus clinical isolates from West Bengal, India: a comparative approach with respect to SA14-14-2 live attenuated vaccine strain
Source: BMC Infect Dis. 2013 Aug 8;13:368. doi: 10.1186/1471-2334-13-368 (PMC3751164; doi:10.1186/1471-2334-13-368)
Supplement: Additional file 1 — Sources of the JEV strains/isolates used in the phylogenetic analysis in this study. All strains/isolates are JEV, except Murray Valley encephalitis virus strain (MVE-1-51) used as an out group for phylogenetic analysis in this study. */** Isolates were 100% identical with each other. [file 1471-2334-13-368-S1.pdf]

Strains/isolates	Country and Year of isolation	Source	Genotype	GenBank Accession no.
Nakayama	Japan, 1935	Human brain	III	U70413
JaGAr01	Japan, 1959	Mosquito	III	AF069076
JaoArS982	Japan, 1982	Mosquito	III	M18370
Ishikawa	Japan, 1998	Mosquito	I	AB051292
JaNAr0102	Japan, 2002	Mosquito	I	AY377577
Fu	Australia,1995	Human	II	AF217620
WTP-70-22	Malaysia ,1970	Mosquito	II	U70421
Muar	Malaysia, 1952	Human	V	HM596272
PhAn1242	Philipines,1984	Pig serum	III	U70417
691004	Srilanka, 1969	Human	III	Z34097
H49778	Srilanka, 1987	Human	III	U70395
DH20	Nepal, 1985	Human	III	U03690
VN118	Vietnam, 1979	Mosquito	III	U70420
K94P05	Korea, 1994	Mosquito	I	AF045551
KV1899	Korea, 1999	Pig	I	AY316157
K91P55	Korea, 1991	Mosquito	I	U34928
GP78	India, 1978	Human	III	AF075723
733913	India, 1973	Human brain	III	Z34095
P20778	India, 1958	Human	III	Z34096
R53567	India, unavailable	unavailable	III	U70418
782219	India, 1982	Human	III	U70402
826309	India, 1982	Human brain	III	U70403
014178	India, 2001	Human blood	III	EF623987
04940-4	India, 2002	Mosquito	III	EF623989
057434	India, 2005	Human blood	III	EF623988
78124	India, 1978	Human	III	U70387
JEV-GKP-0945054	India, 2009	Human CSF	I	HM156572
P3	China, 1949	Mosquito	III	U47032
SA14	China, 1958	Mosquito	III	U14163
SA14-14-2	China, unavailable	Vaccine	III	D90195
GZ04-36	China, 2004	Mosquito	III	DQ404112
XZ0934	China, 2009	Mosquito	V	JF915894
SH-53	China, 2001	Mosquito	I	AY555757
JKT1749	Indonesia, 1979	Mosquito	II	U70405
JKT9092	Indonesia, 1981	Mosquito	IV	U70409
JKT7003	Indonesia, 1981	Mosquito	IV	U70408
JKT5441	Indonesia, 1981	Mosquito	II	U70406
2372	Thailand, 1979	Human	I	U70401
Chiang Mai	Thailand, 1964	Human	III	U70393
HK8256	Taiwan, 1972	Mosquito	III	U03691

Strains/isolates	Country and Year of isolation	Source	Genotype	GenBank Accession no.
IND/11/WB/JEV45	Midnapore, West Bengal, India, 2011	Human CSF	I	KC526872
IND/11/WB/JEV46*	Midnapore, West Bengal, India, 2011	Human CSF	III	KC526869
IND/11/WB/JEV47	Hooghly, West Bengal, India, 2011	Human serum	III	KC526870
IND/11/WB/JEV48*	South 24 Parganas, West Bengal, India, 2011	Human CSF	III	KC802020
IND/11/WB/JEV49*	Howrah, West Bengal, India, 2011	Human serum	III	KC802021
IND/12/WB/JEV50**	Malda, West Bengal, India, 2012	Human CSF	III	KC526871
IND/12/WB/JEV51**	Birbhum, West Bengal, India, 2012	Human serum	III	KC802022
MVEV-1-51	Australia, 1951	Human	-	AF161266

Additional file 1:

All strains/isolates are JEV, except Murray Valley encephalitis virus strain (MVE-1-51) used as an out group for phylogenetic analysis in this study.

*/**Isolates were 100% identical with each other.
