# Supplementary material for: Envelope protein gene based molecular characterization of Japanese encephalitis virus clinical isolates from West Bengal, India: a comparative approach with respect to SA14-14-2 live attenuated vaccine strain
Source: BMC Infect Dis. 2013 Aug 8;13:368. doi: 10.1186/1471-2334-13-368 (PMC3751164; doi:10.1186/1471-2334-13-368)
Supplement: Additional file 2 — (a) Schematic structural representation of E protein of JEV isolates. Here, DI, DII (FL), DIII, ST and TM were symbolized for domain I (red), domain II with orange colored fusion loop (yellow), domain III (blue), stem (magenta) and transmembrane (gray) region respectively including the span/stretch of respective domain/region with above shown numbering, according to previous reports [32, 33]. (b) A comparison of structure-based multiple sequence alignment of JEV isolates-specific E protein with respect to SA14-14-2 vaccine strain. Domains/regions were colored as Figure 2 (a). Secondary structural elements (described in terms of E-extended strand/β-sheet, H-α-helix and C-coil/turn/bend) were shown below each sequence alignment. Underlined bold faces amino acids represent the JEV isolates-specific amino acid substitutions in E protein. Amino acid substitutions leading to escape from antibody neutralization or neutralizing epitopes or related to neuorovirulence/neuroinvasiveness or both found in the JEV isolates with respect to SA14-14-2 vaccine strain were represented by shaded dot, dot and bold asterisk (*) respectively. One amino acid substitution at E158 (Q→P) in the isolate IND/12/WB/JEV50 resulting in secondary structural changes marked as green shaded bold faces. [file 1471-2334-13-368-S2.pdf]

Sl. No.	Amino acid substitutions positions in E protein	HLA-A alleles										
		HLA-A*0101	HLA-A*0201	HLA-A*0202	HLA-A*0203	HLA-A*0206	HLA-A*0301	HLA-A*1101	HLA-A*24	HLA-A*3101	HLA-A*6801	HLA-A*6802
1	N2H	-	-	-	-	-	-	-	-	-	-	-
2	N8H	-	-	-	-	-	-	-	-	-	-	-
3	S64T	-	-	A**T**VTDISTV (40.46)	A**T**VTDISTV (6.62)	A**T**VTDISTV (7.73)	-	**T**VTDISTVA (7.59)	CYHA**T**VTDI (1.512)<br>SYCYHA**T**VT (1.279)	-	-	A**T**VTDISTV (50.35)<br>TVTDISTVA (36.39)
4	N103K	-	-	-	-	-	-	-	GWG**K**GCGLF (1.322)	-	-	-
5	F107L	-	-	-	-	-	-	GNGCG**L**FGK (13.15)	GWGNGCG**L**F (1.257)<br>**L**FGKGSIDT (0.809)	-	-	-
6	S123R	-	-	-	-	-	CAKFSCT**R**K (7.00)	CAKFSCT**R**K (14.09)	KFSCT**R**KAI (0.892)	-	CAKFSCT**R**K (26.92)	-
7	K138E	-	-	-	-	-	-	-	KY**E**VGIFVH (1.251)	-	-	-
8	Q158P	-	-	-	-	-	-	**P**VGASQAAK (5.82)	NYSA**P**VGAS (1.113)	-	-	-
9	V176I	S**I**TLKLGDY (21.78)	-	-	-	-	S**I**TLKLGDY (32.73)	PNAPS**I**TLK (12.25)	-	-	S**I**TLKLGDY (29.92)	TVTPNAPS**I** (9.82)
10	A177T	SI**T**LKLGDY (21.78)	-	-	-	-	SITLKLGDY (32.73)	PNAPSITLK (12.25)	-	-	SITLKLGDY (29.92)	-
11	A222S	-	-	-	-	-	-	-	-	-	-	-
12	G244E	ELLMEFE**E**A (96.61)	ELLMEFE**E**A (6.79)	-	-	-	EFE**E**AHATK (25.47)	-	-	-	-	-
13	G261S	-	-	-	-	-	-	-	-	-	-	-
14	H264Q	-	-	H**Q**ALAGAIV (68.39)	-	**Q**ALAGAIVV (25.76)	-	-	-	-	-	-
15	M279K	-	-	-	-	.	VVEYSSSV**K** (71.29)	VVEYSSSV**K** (3.31)	EYSSSV**K**LT (1.406)	-	-	SV**K**LTSGHL (84.14)
16	E306G	-	-	-	-	-	CT**G**KFSFAK (1.73)	CT**G**KFSFAK (3.18)	GMCT**G**KFSF (1.024)	-	-	-
17	A311R	-	-	-	-	-	CTGKFSF**R**K (1.07)	CTGKFSF**R**K (3.00)	-	-	-	-
18	V315A	-	-	-	-	-	-	-	-	-	-	-
19	S327Q	GTVVIEL**Q**Y (2.87)	-	-	-	-	GTVVIEL**Q**Y (58.88)<br>VIEL**Q**YTGK (0.75)	VIEL**Q**YTGK (10.40)	**Q**YTGKDGPC (1.185)	-	-	-
	S327T	GTVVIEL**T**Y (1.47)	-	-	-	-	GTVVIEL**T**Y (83.95)	VIEL**T**YTGS (18.34)<br>VIEL**T**YSGS (18.34)	**T**YTGSDGPC (1.264)<br>**T**YSGSDGPC (1.18)	-	-	-

Sl. No.	Amino acid substitutions positions in E protein	HLA-A alleles										
		HLA-A*0101	HLA-A*0201	HLA-A*0202	HLA-A*0203	HLA-A*0206	HLA-A*0301	HLA-A*1101	HLA-A*24	HLA-A*3101	HLA-A*6801	HLA-A*6802
20	S329T	-	-	-	-	-	Y**T**GSDGPCK (2.77)<br>Y**T**GKDGPCK (2.70)<br>VIELQY**T**GK (0.75)	Y**T**GSDGPCK (11.25)<br>Y**T**GKDGPCK (11.25)<br>VIELQY**T**GK (10.40)	TYTGSDGPC (1.264)<br>QYTGKDGPC (1.185)	-	-	-
21	S331K	-	-	-	-	-	YTGKDGPCK (2.70)<br>VIELQYTGK (0.75)	YTGKDGPCK (11.25)<br>VIELQYTGK (10.40)	QYTGKDGPC (1.185)	-	-	-
22	A366S	ATSS**S**NSKV (49.66)	ATS**S**SNSKV (8.13)	ATSS**S**NSKV (87.10)	-	ATSS**S**NSKV 7.62)	VATSS**S**NSK (27.35)	VATSS**S**NSK (10.47)	-	-	-	-
23	V372L	-	-	-	-	-	-	-	-	-	-	-
24	M374I	-	-	-	-	-	-	-	-	-	-	-
	G388E	-	-	-	-	-	YIVVGR**E**DK (1.29)	-	-	-	-	VVGR**E**DKQI (6.15)
25	G388K	-	-	-	-	-	YIVVGR**K**DK (0.99)	YIVVGR**K**DK (20.28)<br>DSYIVVGR**K** (18.20)	-	-	-	VVGR**K**DKQI (6.52)
26	D389E	-	-	-	-	-	YIVVGRG**E**K (1.59)	YIVVGRG**E**K (22.03)	-	-	-	VVGRG**E**KQI (7.43)
27	W396R	-	-	-	-	-	-	-	-	-	-	-
28	R416K	-	-	-	-	-	-	-	-	-	-	-
29	G432R	-	-	-	-	-	-	-	-	-	-	-
30	R439K	-	-	-	-	-	-	-	-	-	-	-
31	L463R	-	-	-	-	-	-	-	-	-	-	-
32	L467E	-	-	-	-	-	-	-	-	-	-	-
33	A481D	-	-	-	-	-	-	-	-	-	-	-

Additional file 2

The values given within the bracket indicates the 50% inhibitory concentration (IC50) of the peptide, a measure of the binding affinity. An IC50 value < 50 is considered as a good affinity. Amino acid substitutions in the predicted epitopes of E protein are marked as bold.
