# Supplementary material for: Envelope protein gene based molecular characterization of Japanese encephalitis virus clinical isolates from West Bengal, India: a comparative approach with respect to SA14-14-2 live attenuated vaccine strain
Source: BMC Infect Dis. 2013 Aug 8;13:368. doi: 10.1186/1471-2334-13-368 (PMC3751164; doi:10.1186/1471-2334-13-368)
Supplement: Additional file 3 — Amino acid substitutions in E protein epitopes of JEV WB isolates associated with HLA-A alleles as predicted by EpiJen serve. The values given within the bracket indicates the 50% inhibitory concentration (IC50) of the peptide, a measure of the binding affinity. An IC50 value < 50 is considered as a good affinity. Amino acid substitutions in the predicted epitopes of E protein are marked as bold. [file 1471-2334-13-368-S3.pdf]

Additional file 3

Sl. No.	Amino acid substitutions positions in E protein	HLA-B alleles						
		HLA-B*07	HLA-B*27	HLA-B*40	HLA-B*44	HLA-B*51	HLA-B*53	HLA-B*3501
1	N2H	-	-	-	-	-	-	-
2	N8H	-	-	-	-	-	-	-
3	S64T	-	VRSYCYHA**T** (1.394)	-	-	-	-	-
4	N103K	-	-	-	-	-	-	-
5	F107L	-	-	-	-	CG**L**FGKGSI (76.03)	-	-
6	S123R	-	T**R**KAIGRTI (1.277)	-	-	-	-	-
7	K138E	-	-	-	-	-	-	-
8	Q158P	-	-	-	-	-	-	-
9	V176I	TPNAPS**I**TL (3.11)	-	-	-	TPNAPS**I**TL (62.95)	-	-
10	A177T	TPNAPSI**T**L (3.11)	-	-	-	TPNAPSI**T**L (62.95)	-	-
11	A222S	-	-	REWFHDL**S**L (1.376)	REWFHDL**S**L (1.376)	-	-	-
12	G244E	-	-	-	-	-	-	-
13	G261S	-	-	-	-	-	-	-
14	H264Q	-	-	-	-	**Q**ALAGAIVV (82.79)	-	-
15	M279K	SV**K**LTSGHL (75.86)	-	VEYSSSV**K**L (1.505)	VEYSSSV**K**L (1.505)	-	-	-
16	E306G	-	-	-	-	-	-	-
17	A311R	-	F**R**KNPADTG (1.471)	-	-	-	-	-
18	V315A	-	-	-	-	-	-	-
	S327Q	-	-	-	-	-	-	-
19	S327T	-	-	-	-	-	-	-
20	S329T	-	-	-	-	**T**GSDGPCKI (3.24)	-	-
21	S331K	-	-	-	-	TG**K**DGPCKI (19.41)	-	-
22	A366S	-	-	TSS**S**NSKVL (0.622)	TSS**S**NSKVL (0.622)	-	-	-
23	V372L	-	-	-	-	-	-	-
24	M374I	-	-	-	-	-	-	-
	G388E	-	GR**E**DKQINH (1.656)	-	-	-	-	-
25	G388K	VVGR**K**DKQI (45.19)	GR**K**DKQINH (1.672)	-	-	-	-	-
26	D389E	-	GRG**E**KQINH (1.660)	G**E**KQINHHW (1.094)	G**E**KQINHHW (1.094)	-	-	-
27	W396R	-	-	-	-	-	-	-
28	R416K	-	-	-	-	-	-	-
29	G432R	-	-	-	-	-	-	-
30	R439K	-	-	-	-	-	-	-
31	L463R	-	-	-	-	-	-	-
32	L467E	-	-	-	-	-	-	-
33	A481D	-	-	-	-	-	-	-

The values given within the bracket indicates the 50% inhibitory concentration (IC50) of the peptide, a measure of the binding affinity. An IC50 value < 50 is considered as a good affinity. Amino acid substitutions in the predicted epitopes of E protein are marked as bold.
