# Supplementary material for: Envelope protein gene based molecular characterization of Japanese encephalitis virus clinical isolates from West Bengal, India: a comparative approach with respect to SA14-14-2 live attenuated vaccine strain
Source: BMC Infect Dis. 2013 Aug 8;13:368. doi: 10.1186/1471-2334-13-368 (PMC3751164; doi:10.1186/1471-2334-13-368)
Supplement: Additional file 4 — Amino acid substitutions in E protein epitopes of JEV WB isolates associated with HLA-B alleles as predicted by EpiJen server. The values given within the bracket indicates the 50% inhibitory concentration (IC50) of the peptide, a measure of the binding affinity. An IC50 value < 50 is considered as a good affinity. Amino acid substitutions in the predicted epitopes of E protein are marked as bold. [file 1471-2334-13-368-S4.pdf]

Additional file 4

(a)

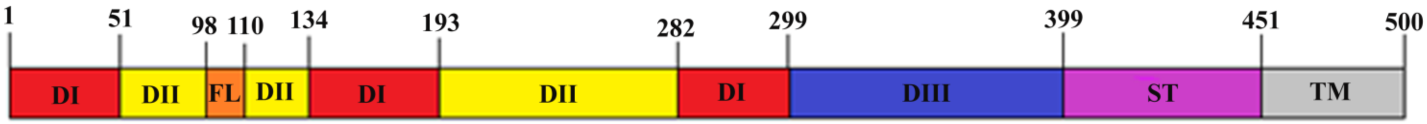

(b)

```
                                                         .                                                                   .     *
KC526872/IND/11/WB/JEV45  FHCLGMGNRDFIEGASGATWVDLVLEGDSCLTIMANDKPTLDVRMINIEASQLAEVRSYC  60
                          CCCCCCCCEEEEECCCCCCEEEEEEECCCEEEEEECCCCEEEEEEEEEEEECCEEEEEEE
KC526869/IND/11/WB/JEV46  FHCLGMGNRDFIEGASGATWVDLVLEGDSCLTIMANDKPTLDVRMINIEASQLAEVRSYC  60
                          CCCCCCCCEEEEECCCCCCEEEEEEECCCEEEEEECCCCEEEEEEEEEEEECCEEEEEEE
KC526870/IND/11/WB/JEV47  FHCLGMGHRDFIEGASGATWVDLVLEGDSCLTIMANDKPTLDVRMINIEASQLAEVRSYC  60
                          CCCCCCCCEEEEECCCCCCEEEEEEECCCEEEEEECCCCEEEEEEEEEEEECCEEEEEEE
KC526871/IND/12/WB/JEV50  FHCLGMGNRDFIEGASGATWVDLVLEGDSCLTIMANDKPTLDVRMINIEASQLAEVRSYC  60
                          CCCCCCCCEEEEECCCCCCEEEEEEECCCEEEEEECCCCEEEEEEEEEEEECCEEEEEEE
         D90195/SA14-14-2 FNCLGMGNRDFIEGASGATWVDLVLEGDSCLTIMANDKPTLDVRMINIEASQLAEVRSYC  60
                          CCCCCCCCEEEEECCCCCCEEEEEEECCCEEEEEECCCCEEEEEEEEEEEECCEEEEEEE

                             .                                     .   *
KC526872/IND/11/WB/JEV45  YHASVTDISTVARCPTTGEAHNEKRADSSYVCKQGFTDRGWGKGCGLFGKGSIDTCAKFS  120
                          EEEEEEEEEEEECCCCCCCCCCHHHHCCCEEEEEEEEEEEECHHHCCCCEEEEEEEEEEE
KC526869/IND/11/WB/JEV46  YHASVTDISTVARCPTTGEAHNEKRADSSYVCKQGFTDRGWGNGCGLFGKGSIDTCAKFS  120
                          EEEEEEEEEEEECCCCCCCCCCHHHHCCCEEEEEEEEEEEECHHHCCCCEEEEEEEEEEE
KC526870/IND/11/WB/JEV47  YHATVTDISTVARCPTTGEAHNEKRADSSYVCKQGFTDRGWGNGCGLFGKGSIDTCAKFS  120
                          EEEEEEEEEEEECCCCCCCCCCHHHHCCCEEEEEEEEEEEECHHHCCCCEEEEEEEEEEE
KC526871/IND/12/WB/JEV50  YHASVTDISTVARCPTTGEAHNEKRADSSYVCKQGFTDRGWGNGCGLFGKGSIDTCAKFS  120
                          EEEEEEEEEEEECCCCCCCCCCHHHHCCCEEEEEEEEEEEECHHHCCCCEEEEEEEEEEE
         D90195/SA14-14-2 YHASVTDISTVARCPTTGEAHNEKRADSSYVCKQGFTDRGWGNGCGFFGKGSIDTCAKFS  120
                          EEEEEEEEEEEECCCCCCCCCCHHHHCCCEEEEEEEEEEEECHHHCCCCEEEEEEEEEEE
```

```
                                                          Glycosylation site
KC526872/IND/11/WB/JEV45 CTSKAIGRTIQPENIKYEVGIFVHGTTTSENHGNYSAQVGASQAAKFTVTPNAPSITLKL 180
                         EEEEEEEEECCHHHEEEEEEEEECCCCCCCCCCCHHHHHHCCCCEEEEECCCCCEEEEEE
KC526869/IND/11/WB/JEV46 CTSKAIGRTIQPENIKYEVGIFVHGTTTSENHGNYSAQVGASQAAKFTVTPNAPSITLKL 180
                         EEEEEEEEECCHHHEEEEEEEEECCCCCCCCCCCHHHHHHCCCCEEEEECCCCCEEEEEE
KC526870/IND/11/WB/JEV47 CTRKAIGRTIQPENIKYEVGIFVHGTTTSENHGNYSAQVGASQAAKFTVTPNAPSITLKL 180
                         EEEEEEEEECCHHHEEEEEEEEECCCCCCCCCCCHHHHHHCCCCEEEEECCCCCEEEEEE
KC526871/IND/12/WB/JEV50 CTSKAIGRTIQPENIKYEVGIFVHGTTTSENHGNYSAPVGASQAAKFTVTPNAPSITLKL 180
                         EEEEEEEEECCHHHEEEEEEEEECCCCCCCCCCCHCHHHCCCCCEEEEECCCCCEEEEEE
      D90195/SA14-14-2 CTSKAIGRTIQPENIKYKVGIFVHGTTTSENHGNYSAQVGASQAAKFTVTPNAPSVALKL 180
                         EEEEEEEEECCHHHEEEEEEEEECCCCCCCCCCCHHHHHHCCCCEEEEECCCCCEEEEEE

KC526872/IND/11/WB/JEV45 GDYGEVTLDCEPRSGLNTEAFYVMTVGSKSFLVHREWFHDLSLPWTSPSSTAWRNRELLM 240
                         CCCEEEEEEEECCCCCCCCCEEEEEECCEEEEEEHHHHHHCCCCEECCCCCCCECHHHCE
KC526869/IND/11/WB/JEV46 GDYGEVTLDCEPRSGLNTEAFYVMTVGSKSFLVHREWFHDLALPWTSPSSTAWRNRELLM 240
                         CCCEEEEEEEECCCCCCCCCEEEEEECCEEEEEEHHHHHHCCCCEECCCCCCCECHHHCE
KC526870/IND/11/WB/JEV47 GDYGEVTLDCEPRSGLNTEAFYVMTVGSKSFLVHREWFHDLALPWTSPSSTAWRNRELLM 240
                         CCCEEEEEEEECCCCCCCCCEEEEEECCEEEEEEHHHHHHCCCCEECCCCCCCECHHHCE
KC526871/IND/12/WB/JEV50 GDYGEVTLDCEPRSGLNTEAFYVMTVGSKSFLVHREWFHDLALPWTSPSSTAWRNRELLM 240
                         CCCEEEEEEEECCCCCCCCCEEEEEECCEEEEEEHHHHHHCCCCEECCCCCCCECHHHCE
      D90195/SA14-14-2 GDYGEVTLDCEPRSGLNTEAFYVMTVGSKSFLVHREWFHDLALPWTSPSSTAWRNRELLM 240
                         CCCEEEEEEEECCCCCCCCCEEEEEECCEEEEEEHHHHHHCCCCEECCCCCCCECHHHCE

KC526872/IND/11/WB/JEV45 EFEEAHATKQSVVALGSQEGSLHQALAGAIVVEYSSSVKLTSGHLKCRLKMDKLALKGTT 300
                         EECCCCCCCCCEEECCCCHHHHHHHCCCCEEECCCCCEECCCCEEEEEEECCCCCCCCCC
KC526869/IND/11/WB/JEV46 EFEEAHATKQSVVALGSQEGSLHQALAGAIVVEYSSSVKLTSGHLKCRLKMDKLALKGTT 300
                         EECCCCCCCCCEEECCCCHHHHHHHCCCCEEECCCCCEECCCCEEEEEEECCCCCCCCCC
KC526870/IND/11/WB/JEV47 EFEEAHATKQSVVALGSQEGSLHQALAGAIVVEYSSSVKLTSGHLKCRLKMDKLALKGTT 300
                         EECCCCCCCCCEEECCCCHHHHHHHCCCCEEECCCCCEECCCCEEEEEEECCCCCCCCCC
KC526871/IND/12/WB/JEV50 EFEEAHATKQSVVALGSQEGSLHHALAGAIVVEYSSSVKLTSGHLKCRLKMDKLALKGTT 300
                         EECCCCCCCCCEEECCCCHHHHHHHCCCCEEECCCCCEECCCCEEEEEEECCCCCCCCCC
      D90195/SA14-14-2 EFEGAHATKQSVVALGSQEGGLHHALAGAIVVEYSSSVMLTSGHLKCRLKMDKLALKGTT 300
                         EECCCCCCCCCEEECCCCHHHHHHHCCCCEEECCCCCEECCCCEEEEEEECCCCCCCCCC
```

```
                          *    .   .          .  .  .
KC526872/IND/11/WB/JEV45 YGMCTEKFSFAKNPADTGHGTVVIELTYSGSDGPCKIPIVSVASLNDMTPVGRLVTVNPF 360
                         CCECCCCEEEEEEEEECCCCCEEEEEEEECCCCCCEECCEEEEECCCCCCCCCEECCCCCE
KC526869/IND/11/WB/JEV46 YGMCTGKFSFRKNPADTGHGTVVIELTYTGSDGPCKIPIVSVASLNDMTPVGRLVTVNPF 360
                         CCECCCCEEEEEEEEECCCCCEEEEEEEECCCCCCEECCEEEEECCCCCCCCCEECCCCCE
KC526870/IND/11/WB/JEV47 YGMCTGKFSFAKNPADTGHGTVVIELQYTGKDGPCKIPIVSVASLNDMTPVGRLVTVNPF 360
                         CCECCCCEEEEEEEEECCCCCEEEEEEEECCCCCCEECCEEEEECCCCCCCCCEECCCCCE
KC526871/IND/12/WB/JEV50 YGMCTEKFSFAKNPVDTGHGTVVIELSYSGSDGPCKIPIVSVASLNDMTPVGRLVTVNPF 360
                         CCECCCCEEEEEEEEECCCCCEEEEEEEECCCCCCEECCEEEEECCCCCCCCCEECCCCCE
       D90195/SA14-14-2 YGMCTEKFSFAKNPVDTGHGTVVIELSYSGSDGPCKIPIVSVASLNDMTPVGRLVTVNPF 360
                         CCECCCCEEEEEEEEECCCCCEEEEEEEECCCCCCEECCEEEEECCCCCCCCCEECCCCCE

                              .                       ..
KC526872/IND/11/WB/JEV45 VATSSANSKVLLEIEPPFGDSYIVVGRKDKQINHHRHKAGSTLGKAFSTTLKGAQRLAAL 420
                         ECCCCCCCEEEEEEECCCEEEEEEEECCCCCCEEEEEEEECCCCHHHHHHHHHHHHHHHHC
KC526869/IND/11/WB/JEV46 VATSSSNSKVLVEMEPPFGDSYIVVGRGEKQINHHWHKAGSTLGKAFSTTLKGAQRLAAL 420
                         ECCCCCCCEEEEEEECCCEEEEEEEECCCCCCEEEEEEEECCCCHHHHHHHHHHHHHHHHC
KC526870/IND/11/WB/JEV47 VATSSSNSKVLVEMEPPFGDSYIVVGREDKQINHHWHKAGSTLGKAFSTTLKGAQKLAAL 420
                         ECCCCCCCEEEEEEECCCEEEEEEEECCCCCCEEEEEEEECCCCHHHHHHHHHHHHHHHHC
KC526871/IND/12/WB/JEV50 VATSSANSKVLVEMEPPFGDSYIVVGRGDKQINHHWHKAGSTLGKAFSTTLKGAQKLAAL 420
                         ECCCCCCCEEEEEEECCCEEEEEEEECCCCCCEEEEEEEECCCCHHHHHHHHHHHHHHHHC
       D90195/SA14-14-2 VATSSANSKVLVEMEPPFGDSYIVVGRGDKQINHHWHKAGSTLGKAFSTTLKGAQRLAAL 420
                         ECCCCCCCEEEEEEECCCEEEEEEEECCCCCCEEEEEEEECCCCHHHHHHHHHHHHHHHHC

                                           .
KC526872/IND/11/WB/JEV45 GDTAWDFGSIGGVFNSIGKAVHQVFGGAFRTLFGGMSWITQGLMGALLLWMGVNARDRSI 480
                         CCCCECCCCCCCHHHHHHHHHHHHHHHHHHHHHCCCCHHHHHHHHHHHHHHHCCCCCCCCC
KC526869/IND/11/WB/JEV46 GDTAWDFGSIGGVFNSIGKAVHQVFGGAFRTLFGGMSWITQGRMGALLLWMGVNARDRSI 480
                         CCCCECCCCCCCHHHHHHHHHHHHHHHHHHHHHCCCCHHHHHHHHHHHHHHHCCCCCCCCC
KC526870/IND/11/WB/JEV47 GDTAWDFGSIGRVFNSIGKAVHQVFGGAFRTLFGGMSWITQGLMGAELLWMGVNARDRSI 480
                         CCCCECCCCCCCHHHHHHHHHHHHHHHHHHHHHCCCCHHHHHHHHHHHHHHHCCCCCCCCC
KC526871/IND/12/WB/JEV50 GDTAWDFGSIGGVFNSIGRAVHQVFGGAFRTLFGGMSWITQGLMGALLLWMGVNARDRSI 480
                         CCCCECCCCCCCHHHHHHHHHHHHHHHHHHHHHCCCCHHHHHHHHHHHHHHHCCCCCCCCC
       D90195/SA14-14-2 GDTAWDFGSIGGVFNSIGRAVHQVFGGAFRTLFGGMSWITQGLMGALLLWMGVNARDRSI 480
                         CCCCECCCCCCCHHHHHHHHHHHHHHHHHHHHHCCCCHHHHHHHHHHHHHHHCCCCCCCCC
```

KC526872/IND/11/WB/JEV45	ALAFLATGGVLVFLATNVHA	500
	EEEEHHHHHEEEEEEEECCCC	
KC526869/IND/11/WB/JEV46	ALAFLATGGVLVFLATNVHA	500
	EEEEHHHHHEEEEEEEECCCC	
KC526870/IND/11/WB/JEV47	**D**LAFLATGGVLVFLATNVHA	500
	EEEEHHHHHEEEEEEEECCCC	
KC526871/IND/11/WB/JEV50	ALAFLATGGVLVFLATNVHA	500
	EEEEHHHHHEEEEEEEECCCC	
D90195/SA14-14-2	ALAFLATGGVLVFLATNVHA	500
	EEEEHHHHHEEEEEEEECCCC	
